# Dynamic Edge Convolutional Neural Network for Skeleton-Based Human Action Recognition

**DOI:** 10.3390/s23020778

**Published:** 2023-01-10

**Authors:** Nusrat Tasnim, Joong-Hwan Baek

**Affiliations:** School of Electronics and Information Engineering, Korea Aerospace University, Goyang 10540, Republic of Korea

**Keywords:** human action recognition, attention mechanism deep learning, edge convolution, dynamic graph update

## Abstract

To provide accessible, intelligent, and efficient remote access such as the internet of things, rehabilitation, autonomous driving, virtual games, and healthcare, human action recognition (HAR) has gained much attention among computer vision researchers. Several methods have already been addressed to ensure effective and efficient action recognition based on different perspectives including data modalities, feature design, network configuration, and application domains. In this article, we design a new deep learning model by integrating criss-cross attention and edge convolution to extract discriminative features from the skeleton sequence for action recognition. The attention mechanism is applied in spatial and temporal directions to pursue the intra- and inter-frame relationships. Then, several edge convolutional layers are conducted to explore the geometric relationships among the neighboring joints in the human body. The proposed model is dynamically updated after each layer by recomputing the graph on the basis of k-nearest joints for learning local and global information in action sequences. We used publicly available benchmark skeleton datasets such as UTD-MHAD (University of Texas at Dallas multimodal human action dataset) and MSR-Action3D (Microsoft action 3D) to evaluate the proposed method. We also investigated the proposed method with different configurations of network architectures to assure effectiveness and robustness. The proposed method achieved average accuracies of 99.53% and 95.64% on the UTD-MHAD and MSR-Action3D datasets, respectively, outperforming state-of-the-art methods.

## 1. Introduction

Over the past years, the utilization of electronic devices such as smartphones, digital television, personal computers, smart elevators, drones, and many others has increased dramatically owing to their affordable cost and the demands of work. The user interest in these devices can be enhanced by introducing vision-based interaction. Vision-based interaction, particularly human action recognition (HAR), plays a vital role in providing easy, smart, and comfortable communication with these devices through various applications such as human–machine or human–object interaction [1], rehabilitation [2], video surveillance systems [3], augmented/virtual reality [4,5], healthcare systems [6], autonomous driving systems [7], and virtual games [8]. Currently, the need for accurate and efficient vision-based communication is increasing significantly owing to the appearance of the COVID-19 pandemic, which has led to the avoidance of intimate contact with devices. Unlike traditional wireless mouse and keyboards, vision-based interaction allows controlling electronic devices without touching any part of them, in addition to providing effective remote control.

**Figure 1:**
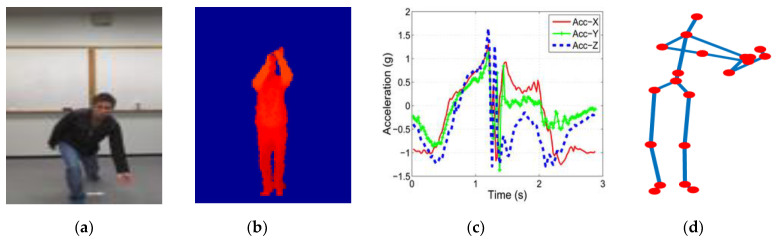
Different modalities of datasets: (**a**) RGB, (**b**) depth, (**c**) inertial, and (**d**) skeleton datasets.

To provide effective and efficient HAR methods, much research has been conducted on different data modalities. The most used data modalities in HAR are RGB, depth, inertial, and skeleton datasets. The dataset varies according to action types, modalities, views, interaction types, etc. The RGB format datasets contain rich information that explicitly provides a clear object structure, as well as background information. Several methods have studied RGB datasets [9,10] and built many effective and efficient HAR methods. However, the RGB format datasets require a larger storage capacity to handle and much computation to perform action recognition because they provide three channels of information. Compared to the RGB datasets, depth format datasets [11,12,13] contain less information with one channel but have enough information to perform action classification. They need less storage capacity, and simple algorithms can process the dataset to recognize the action. A few methods have estimated depth from RGB sequences and recognized human action. Sahoo et al. [14] proposed human action recognition using sequential learning with deep bidirectional long short-term memory and shape learning with depth history images. Even though depth datasets contain one-channel information, they provide object structure and background information with two different colors, making them confusing and time-consuming to process for lightweight devices. Inertial datasets [15] provide more compact information for human action. They contain acceleration and gyroscope information along the x-, y-, and z-axes, which can be processed easily. In addition, the problem with inertial datasets is that we must wear the inertial device on our body to capture action information. It is sometimes cumbersome to wear inertial sensor devices. Consequently, many researchers have concentrated on building skeleton-based HAR methods. Skeleton datasets provide 3D joint information of the human body along the x-, y-, and z-axes, which are very concise, easy to handle, and easily processable. Compared to the RGB, depth, and inertial datasets, 3D skeleton datasets are much better in terms of storage and computation, which inspired us to use them for the proposed method to recognize human action. Figure 1 shows examples of the four data modalities used for HAR methods: (a) RGB, (b) depth, (c) inertial (acceleration), and (d) skeleton data modalities.

The compact representation of human action sequences in skeleton format has facilitated the development of several effective HAR methods. In this article, we analyze the 3D skeleton datasets, attention mechanisms, and deep learning to introduce a novel approach for HAR. The contributions of the proposed study are summarized as follows:

1. We analyzed the 3D skeleton datasets and found that the original joints, as well as the velocity between neighboring frames, have a great impact on the performance of HAR methods. Consequently, we used both original joints and velocity to build a new HAR method.
2. We studied edge convolution to design a novel deep learning model called the dynamic edge convolution neural network to recognize human action. The proposed deep learning model is updated dynamically after each layer by recomputing the graph for each joint using k-nearest neighbors.
3. We also explored criss-cross attention (CCA) before applying edge convolution to emphasize intra- and inter-frame relationships in the spatial and temporal directions.
4. The proposed method was evaluated on two benchmark human action datasets to show its effectiveness and robustness.
5. In addition, we provide extensive experimental results for original joints and velocity, as well as spatial and temporal attention, to show the effects of each component on the performance.

The remainder of this article is organized as follows: Section 2 provides preliminaries that are important to understand the proposed method. In Section 3, we add a brief review of model-based and learning-based HAR methods. Section 4 includes an illustration of the proposed method. Section 5 shows the experimental details by evaluating and comparing the proposed method with the state-of-the-art methods. Lastly, Section 6 integrates the conclusion of the article.

## 2. Preliminaries

This section covers the preliminary knowledge required for the proposed HAR method. We provide overviews of traditional and graph convolution operations, as well as attention mechanisms, with necessary figures.

### 2.1. Traditional Convolution Layer

Generally, convolution refers to a filtering operation in which a filter is applied to an input to generate new feature maps. Standard convolution operation [16] performs multiplication and summation operations on the input with a filter along the spatial and temporal directions having stride and padding information. For example, we have a 2D image with height, width, and channel as H, W, and C, and a kernel (W) of size $3\times 3\times C^{\prime }$ is applied to the 2D image. Then, it produces an output having height, width, and channel as $H^{\prime }$, $W^{\prime }$, and $C^{\prime }$ as the weighted average of the input and kernel.

**Figure 2:**
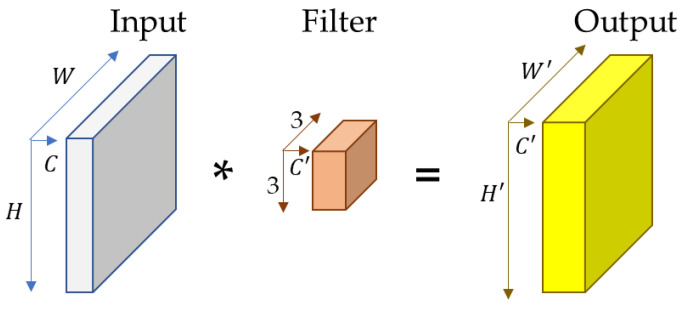
Traditional convolution operation with kernel size of $3\times 3\times C^{\prime }$, where $C^{\prime }$ indicates the output dimension of the feature maps.

The kernel is initialized using different functions based on the size of the input data which can be 1D, 2D, or 3D. Figure 2 shows an example of a standard convolution operation. Traditional convolution operation operates on ordered and structured data, which limits it to processing unordered and unstructured data such as graphs, specifically human skeleton joints graphs. In traditional convolution operation, the same size of the kernel is applied to the whole input data.

### 2.2. Graph Convolution

**Figure 3:**
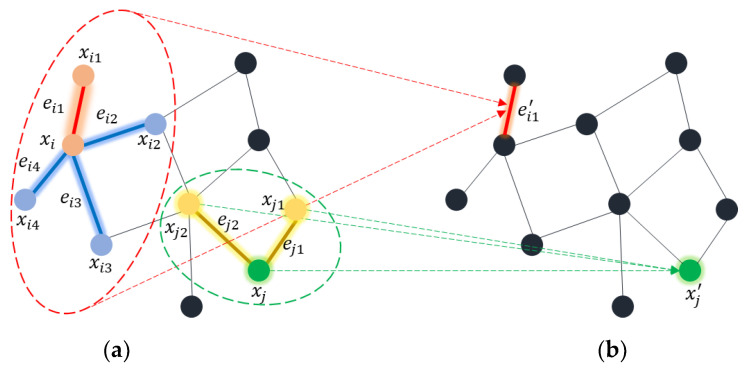
Graph convolution operations: green and red dotted circles represent (**a**) node and edge convolution operations neighbors on the **left** side and (**b**) convolution outputs on the **right** side.

The definition of a graph involves vertices and edges in which vertices and edges represent the nodes and lines between two nodes. Graph convolution can be performed in a node-based or edge-based fashion [17]. Figure 3 illustrates the basic principles of node and edge convolution operations. Node convolution operates on nodes to extract high-level features among the nodes. On the other hand, edge convolution involves exploring the relationships among the edges. For examples, we have a graph $G=\left(V,\;E\right)$, where V= {$v_{i};i=1,\;2,\;\ldots ,\;N$} and E= {$e_{ij};v_{i},v_{j}\;are\;adjacent\;nodes$}. N indicates the total number of nodes in the graph.

To perform node convolution at node $x_{j}$ in Figure 3a, the weighted average of neighboring nodes such as $x_{j1}$ and $x_{j2}$ is determined as shown in Figure 3b with green color. Similarly, to accomplish edge convolution at edge $e_{i1}$ in Figure 3a, the aggregation of edge features with all edges, $e_{i2}$, $e_{i3}$, and $e_{i4}$ formed by neighboring nodes $x_{i1}$, $x_{i2}$, $x_{i3}$, and $x_{i4}$ is computed as shown in Figure 3b.

### 2.3. Attention Mechanisms

**Figure 4:**
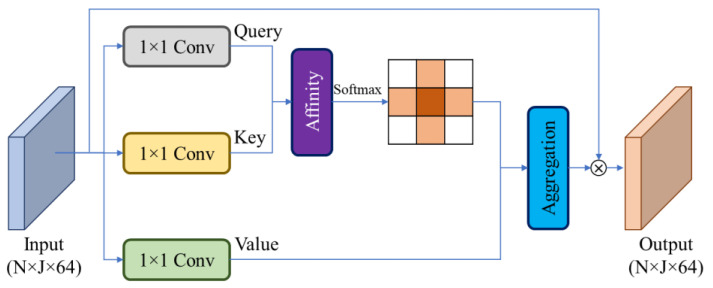
Criss-cross attention mechanism for the proposed HAR method.

Attention refers to a deep concentration on a certain property by slacking others when operating a huge amount of information. In deep learning, attention mechanisms are widely used for various purposes such as machine translation, visual attention, vision transformer, recognition, and self-driving. Several methods have studied attention techniques for human action recognition [18,19]. Most of them focused on capturing local and global relationships among frames by integrating attention methods. Compared to conventional attention, CCA requires less computation [20], and it can obtain the same contextual information as conventional attention. It accomplishes sparse operations among surrounding data along vertical and horizontal directions. Figure 4 shows the working principles of the CCA mechanism, which can capture long-distance relationships. For the proposed method, the input to the CCA module is a feature map extracted by convolution blocks size of N×J×64, where *N* represents number of frames in an action and *J* indicates the number of joints in a frame. The CCA module performs three 1×1 convolutions to generate three feature maps called query (Q), key (K), and Value (V). After obtaining ***Q*** and ***K*** feature maps, an affinity operation is applied to generate attention maps, defined as follows:

(1) $$d_{i,\;x}=Q_{x}\Omega_{i,\;x},$$

where $d_{i,\;x}$ is the degree of freedom between features $Q_{x}$ for Q and set $\Omega_{i,\;x}$ for features K; i=[1,…, H+W−1] at spatial position x. H and W are the height and width of the feature maps.

Then, we apply softmax along the channel dimension to compute the attention map (A). The output feature map from the softmax layer and ***V*** feature map are then aggregated to capture contextual information, defined as follows:

(2) $$Output_{x}=\overset{H+W-1}{\underset{i=0}{\sum }}A_{i,x}\phi_{i,x}+Input_{x},$$

where $A_{i,x}$ is a value at element i in position x. $\phi_{i,x}$ is set in feature $V_{x}$, defined in [20].

Lastly, element-wise multiplication is performed between the input and aggregated feature maps to generate output feature maps.

Attention modules in HAR methods are integrated to obtain intra- and inter-frame relationships for certain information, such as spatial and temporal. In the proposed method, first, we apply an attention module in the spatial domain to capture the local relationship among the joints. Spatial attention focuses on exploring the relationship among 20 joints in a frame. Then, we use an attention module in the temporal domain to understand the global connection among the frames. The temporal attention module finds the attention map of each frame temporally without considering spatial relationships.

## 3. State-of-the-Art Methods

Among the diverse data modalities such as RGB, depth, inertial, and skeleton, the skeleton dataset is more demanding and effective for HAR methods. RGB, depth, and inertial datasets have several disadvantages, as described in Section 1. Recent research has mainly focused on building HAR methods for remote access control, which is integrated with lightweight devices such as mobile phones. Consequently, RGB, depth, and inertial data formats are hard to handle. Because skeleton data formats provide more compact information with only 3D coordinate values, they are easily manageable and can be used to build HAR methods for lightweight devices. The upcoming subsections describe state-of-the-art HAR methods introduced using 3D skeleton data modalities, as listed in Table 1 in terms of category, year of publication, method, and dataset [21].

### 3.1. Traditional Machine Learnning-Based Approaches for HAR Using 3D Skeleton Datasets

**Figure 5:**
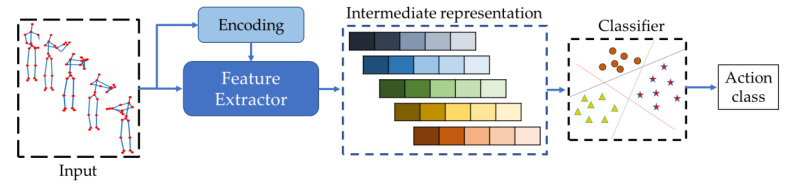
Working scenario of traditional machine learning-based HAR methods.

Traditional machine learning-based HAR methods focus on the intermediate representation of the raw skeleton sequences and apply multiclass classifiers for action recognition, as shown in Figure 5. The raw skeleton sequences can sometimes be encoded to spatiotemporal images [22,23,24,25,26,27,28,40], and then discriminative features are extracted using feature descriptors. The encoding is conducted to enhance the discriminability of the feature maps by keeping intra- and inter-frame relationships. Yang et al. [22] analyzed the intrinsic relationship between joint configurations and action classes to extract discriminative features and used multi-instance multitask learning (MIMTL) for action recognition. Hussein et al. [23] applied the covariance of 3D joints (Cov3DJ) descriptor to understand the changes in skeleton joint locations in the temporal direction. They operated over subsequences with fixed length for action classification using a support vector machine (SVM). Xia et al. [24] extracted histograms of 3D joint locations (HOJ3D) and projected them using linear discriminant analysis (LDA) to cluster into k postures for HAR. They applied the hidden Markov model (HMM) for action recognition. Yang et al. [25] proposed a new feature set including position differences of joints and eigenjoints by considering static posture, temporal changes, and offset. They trained naïve Bayes nearest neighbors (NBNN) classifiers to recognize action sequences. Vemulapalli et al. [26] explored the geometric relationships by segmenting joints of the human body skeleton with rotation and translation along the x-, y-, and z-axes. Agahian et al. [27] introduced a new feature descriptor consisting of normalized 3D skeleton coordinates, inter-joints, and inter-frame changes. They trained an SVM and extreme learning model (ELM) with the proposed feature for action recognition. Chaudhry et al. [28] proposed discriminative metrics using linear dynamic systems (LDS) of spatiotemporal changes with different scales and learned a set of optimal weights using multiple kernel learning (MKL). Lv et al. [40] converted the whole 3D skeleton sequence into a feature set containing temporal changes for single and multiple joints and used a multiclass Adaboost classifier for action recognition.

### 3.2. Deep Learning-Based Approaches for HAR Using 3D Skeleton Datasets

**Figure 6:**
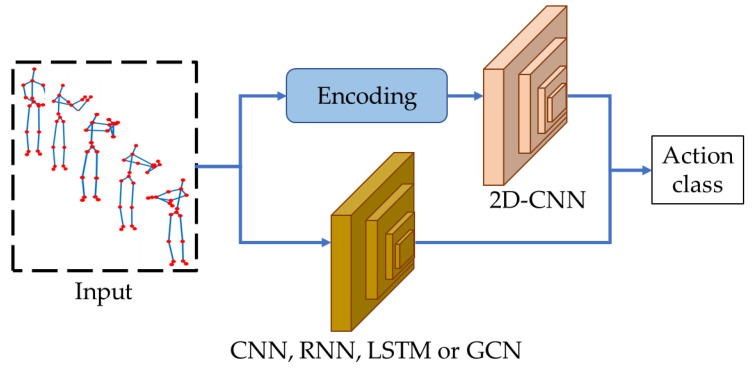
Working scenario of deep learning-based HAR methods.

Because traditional machine learning-based approaches require an intermediate representation of raw skeleton sequences and applied multiclass classifiers for action classifications, they are time-consuming and need user intervention. The performance of the systems is fully confined to the effective intermediate representations. Consequently, deep learning-based methods [29,30,31,32,33,34,35,36,37,38,39,40] have become very popular for HAR as shown in Figure 6. Deep learning-based methods focus on the extraction and recognition of human action sequences directly without any user assistance.

Sometimes, the raw skeleton sequences are encoded in spatiotemporal format [29,30,31,32] for better representation, called skeleton optical spectra (SOS), and then applied deep learning-based methods for action recognition. The spatiotemporal encoding of 3D skeleton joints into spatiotemporal images is accomplished with different color models such as RGB and HSB to capture spatial and temporal changes. Hou et al. [29] introduced an encoding-based HAR method in which they represented the 3D skeleton joints with different colors in the temporal directions in three cartesian planes and formed three-pixel domain skeleton optical spectra (SOS) images. They applied three branches of convolutional networks (ConvNets) for the front, top, and side views of optical spectra images and fused them for action classification. A similar approach was proposed by Wang et al. [30] to encode 3D skeleton joints into three spatial formats called joint trajectory maps (JTMs). They projected 3D skeleton joints onto three cartesian planes and captured motion direction by representing different segments of joints with different colors along the temporal direction. They encoded the joint trajectory maps in HSB color format and then converted them into RGB color format to train ConvNets for action recognition. Chen et al. [31] studied the previous spatiotemporal representation and found that the encoding technique cannot maintain long-term temporal changes. They introduced a new encoding method, temporal pyramid skeleton motion maps (TPSMMs), to convert 3D skeleton sequences into color texture images. They partitioned the input sequence into segments to generate size spatiotemporal images and conducted size branches of ConvNets for feature extraction and classification. Tasnim et al. [32] studied spatiotemporal encoding of 3D skeleton joints and introduced a method called spatiotemporal image formation (STIF) using a jet color model. They presented the joints and lines between joints with different colors to capture temporal changes and used deep learning models for action recognition.

The encoding techniques are fully dependent on the effective representation of the spatiotemporal images. The encoded images cannot maintain full temporal information and degrade the performance of the HAR methods. Furthermore, the intermediate representation is time-consuming. Consequently, the usage of raw 3D skeleton joints for HAR is important. Several approaches have adopted raw 3D skeleton joints for action recognition using a convolutional neural network (CNN) [33] and graph convolutional network (GCN) [34,35,36,37,38,39,41]. Wang et al. [33] investigated the inter-frame changes on the basis of edges and joints and used edge motion and joint location branches with heatmap branches to propose a skeleton edge motion network (SEMN) for action recognition. Zhao et al. [34] considered spatial dependencies among joints, temporal variations, and action execution for HAR using a GCN and long-short term memory (LSTM). Ahmad et al. [35] proposed a method for HAR based on graph sparsification by removing unnecessary nodes and edge information using attention and a spatiotemporal GCN (ST-GCN) model. Liu et al. [36] built a skeleton graph based on spatial structure and temporal evaluation and passed through three GCN branches called adaptive multi-view GCN (AMV-GCN). Two of them were rotated clockwise and anticlockwise and fused with the original branch to recognize action. Liu et al. [37] extended the ST-GCN and added ResNeXt for HAR using 3D skeleton joint information. They constructed a spatiotemporal graph and skeleton motion image from raw skeleton joints and passed through two branches for extracting discriminative features for action classification. Zhang et al. [38] selected key nodes and frames to reduce complexity of GCN and applied scaling, rotation, and translation to improve the performance. Cha et al. [39] reconstructed the RGB data into 3D meshes and explored intra- and inter-structural relationships of 3D meshes to recognize human action using the transformer method. Wu et al. [41] proposed multimodal human action recognition using hierarchical pyramid depth motion images (HP-DMI) and spatiotemporal joint descriptors (STJD). Then, they extracted a histogram of oriented gradient (HOG) features from HP-DMI and named it HP-DMI-HOG. An SVM was conducted to recognize the action classes.

## 4. Proposed Methodology

This section concentrates on the illustration of the proposed method for HAR including the architectural overviews, preprocessing of 3D skeleton information, and dynamic graph updates in the proposed model.

### 4.1. Architectural Overviews of the Proposed Method

**Figure 7:**
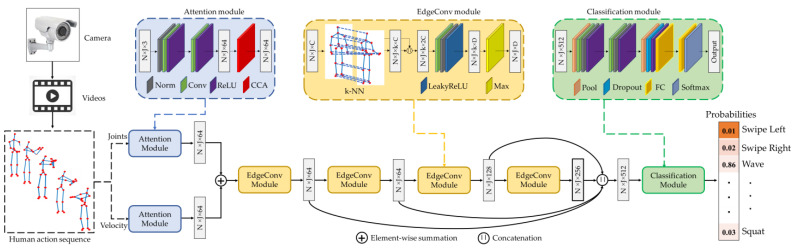
Overall working principle of the proposed dynamic edge convolutional neural network for human action recognition.

Figure 7 shows the overall block diagram of the proposed method. First, a camera is used to capture video sequences in terms of 3D skeleton joint information of the human body, which is then processed by several modules to perform the action classification. The proposed method has three major modules: attention, edge convolution (EdgeConv), and classification modules. We applied attention modules separately for original joints and velocity. Before applying the CCA attention techniques, we conducted a normalization, two 1×1 convolutions, and a rectified linear unit (ReLU) layer whtoich convert the input information from N×J×3 to N×J×64 feature maps. We conducted a criss-cross attention module to emphasize spatial and temporal relationships. The feature maps generated from two attention modules are then summed and passed through edge convolution blocks. Four edge convolution layers are conducted sequentially, in which each layer contains a k-NN graph, a convolution, a normalization, a LeakyReLU, and a max layer. The four subsequent edge convolution layers progressively generate 64-, 64-, 128-, and 256-dimensional features. The feature maps are concatenated to form a 512-dimensional feature map and classified using a classification module. The classification module consists of four blocks. The first block contains an adaptive max pooling layer, a convolution layer, a normalization layer, and a ReLU layer. The second block consists of a convolution layer, a normalization layer, and a ReLU layer. The third block has an adaptive max pooling layer, a dropout layer, a ReLU layer, and a fully connected layer. Lastly, the fourth block contains a fully connected layer and a softmax layer. The softmax layer converts the feature maps into probability maps to decide the output class.

### 4.2. Preprocessing of 3D Skeleton Joints and Dynamic Graph Updates

Among the diverse 3D skeleton-based HAR methods, several methods have shown that the usage of velocity along with original joints greatly improves the overall performance of the system. Consequently, we consider both the original 3D skeleton joints and the velocity between the neighboring joints. Let us assume that $f_{i,j}$ and $f_{i+1,j}$ are the current and next frames in an action sequence, where i=1, 2, …, N is the number of frames, and j=1, 2, …, 20 is the number of joints. Then, the velocity at the current frame ($v_{i,j}$) is the difference between the current and next frames, defined as follows:

(3) $$v_{i,j}=f_{i+1,j}-f_{i,j}.$$

The original joints ($f_{i,j}$) and velocity ($v_{i,j}$) were used for the proposed method to recognize human action, as shown in Figure 7. The dynamic edge convolutional neural network has been successfully implemented in point cloud processing such as classification, segmentation [42], and incomplete region estimation [43]. For the skeleton space, we order the joints along the temporal direction. Then, the pairwise Euclidean distances are computed between two joints, and k-nearest neighbor joints are chosen to build a k-NN graph on the basis of the minimum distance in the same frame or across frames. This generates an edge feature set of size k for each joint at each EdgeConv layer, and the max layer is applied to neighboring edges to produce a feature map of shape N×J×D.

Unlike PointNet [44], PointNet++ [45], and the graph convolutional neural network, which operate on a fixed graph, the dynamic edge convolutional neural network is updated dynamically after each layer by recomputing the graph of neighbors in the feature domain. The recomputing of the graph after each layer in the feature domain helps in choosing the best arrangement of the graph with k-nearest neighbor joints. The dynamic graph computation in each layer can be formulated as follows:

(4) $$G^{i}=\left(V_{i},\;E_{i}\right);\;\;\;\;\;\;\;i=1,\;2,\;3,\;4,$$

where $G^{i}$ indicates the number of graphs in the proposed model. Because we used four edge convolution layers, a total of four graphs were computed. $V_{i}$ and $E_{i}$ represent the vertex and edge sets in each layer, which vary according to the intermediate feature representation.

Compared to PointNet, PointNet++, and graph convolution, dynamic edge convolution is permutation- and translation-invariant [42]. This is because dynamic edge convolution aggregates features by applying a symmetric function such as global max pooling.

## 5. Experimental Results

This section demonstrates the experimental environments, performance evaluation and comparisons, and complexity of the proposed model for human action recognition using 3D skeleton information.

### 5.1. Experimental Setup

We accomplished the experiments on Ubuntu 20.04 operating system with a GeForce GTX 1080Ti GPU. We adopted the PyTorch framework in python programming for designing the proposed deep learning model for HAR.

Different parameter settings were conducted to show the effectiveness and robustness of the proposed deep learning model. We trained the proposed model for 500 epochs with a learning rate of 0.001. The batch size was set to 64, and the model was optimized using stochastic gradient descent (SGD) [46]. The learning rate was reduced by 10% after every 100 epochs. We used a cross-entropy loss function for training the proposed model.

### 5.2. Evaluation Metrics

Because the MSR-Action3D dataset contains imbalanced samples, we computed precision, recall, accuracy, and F1-score for performance evaluation. TP, TN, FP, and FN are the true positive, true negative, false positive, and false negative samples; thus, precision, recall, accuracy, and F1-score can be defined as follows:

(5) $$Precision\;\left(\%\right)=\frac{TP}{TP+FP}\times 100,$$

(6) $$Recall\;\left(\%\right)=\frac{TP}{TP+FN}\times 100,$$

(7) $$Accuracy\;\left(\%\right)=\frac{TP}{TP+TN+FP+FN}\times 100,$$

(8) $$F1\;Score\;\left(\%\right)=\frac{2\times TP}{2\times TP+FP+FN}\times 100$$

Because of the limited number of training samples, we applied data augmentation. We conducted rotation and transformation augmentation techniques.

### 5.3. Dataset Descriptions

We applied the proposed method to two 3D skeleton benchmark datasets: UTD-MHAD (University of Texas at Dallas multimodal human action dataset) [47] and MSR-Action 3D (Microsoft action 3D) [48].

**Figure 8:**
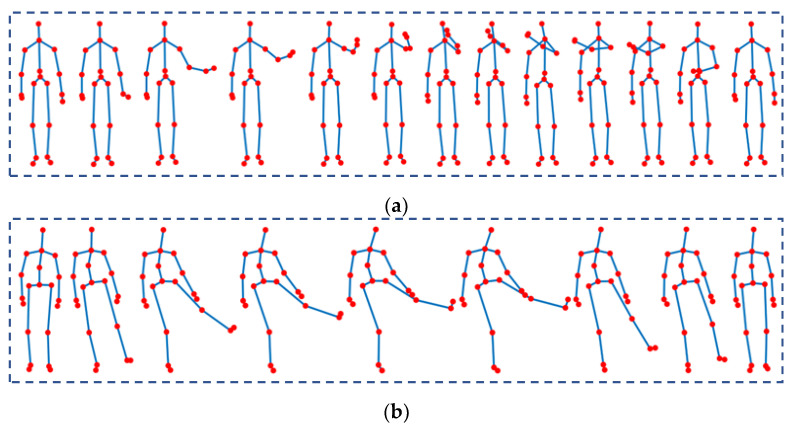
Examples of actions in skeleton datasets: (**a**) *SwipeLeft* in UTD-MHAD dataset; (**b**) *SideKick* in MSR-Action3D dataset.

A group of researchers captured the UTD-MHAD dataset at the embedded system and signal processing laboratory at the University of Texas in Dallas. This dataset contains 27 action categories: *SwipeLeft*, *SwipeRight*, *Wave*, *Clap*, *Throw*, *ArmCross*, *BasketballShoot*, *DrawX*, *DrawCircle* (*CLW*), *DrawCircle* (*CCLW*), *DrawTriangle*, *Bowling*, *Boxing*, *BaseballSwing*, *TennisSwing*, *ArmCurl*, *TennisServe*, *Push*, *Knock*, *Catch*, *PickUpThrow*, *Jog*, *Walk*, *SitToStand*, *StandToSit*, *Lunge*, and *Squat*. Each action has 32 sequences performed by eight individuals. There are three corrupted data, which provide a total of 861 sequences. Figure 8a shows the *SwipeLeft* action sequence in the UTD-MHAD dataset.

Wanqing Li and the communication and collaboration systems research group at Microsoft Research Red-Mond captured the MSR-Action3D dataset. This dataset contains 20 action categories: *HighArmWave*, *HoizontalArmWave*, *Hammer*, *HandCatch*, *ForwardPunch*, *HighThrow*, *DrawX*, *DrawTick*, *DrawCircle*, *HandClap*, *TwoHandWave*, *SideBoxing*, *Bend*, *ForwardKick*, *SideKick*, *Jog*, *TennisSwing*, *TennisServe*, *GolfSwing*, and *PickUpandThrow*. There are a total of 567 action sequences performed by 10 different individuals. The first, second, and third classes have 27 samples. The fourth, fifth, and sixth classes have 26 samples. The eighth and 15th classes have 28 and 20 samples, respectively. The remaining classes have 30 samples each. Figure 8b shows the *SideKick* action sequence in the MSR-Action3D dataset.

We followed the same data partitioning as described in [27]. We used the dataset captured by odd and even individuals for training and testing, respectively. For the UTD-MHAD dataset, we trained and tested the proposed method with 431 and 430 action sequences. For the MSR-Action3D dataset, we trained and tested the proposed method with 292 and 275 action sequences.

### 5.4. Performance Evaluations and Comparisons on the UTD-MHAD Dataset

We evaluated the proposed method with original joints (joints), the difference between neighboring joints (velocity), the joints and velocity (joints + velocity), and the joints and velocity with CCA (joints + velocity + CCA) in the spatial and temporal domains. Table 2 lists the recognition precision, recall, accuracy, and F1-score on the UTD-MHAD dataset. The proposed method secured approximately 98.84%, 96.51%, 99.07%, and 99.53% recognition accuracies for joints, velocity, joints + velocity, and joints + velocity + CCA, respectively. The best setting (joints + velocity + CCA) improved the performance by approximately +0.69%, +3.02%, and +0.46% compared to joints, velocity, and joints + velocity.

**Figure 9:**
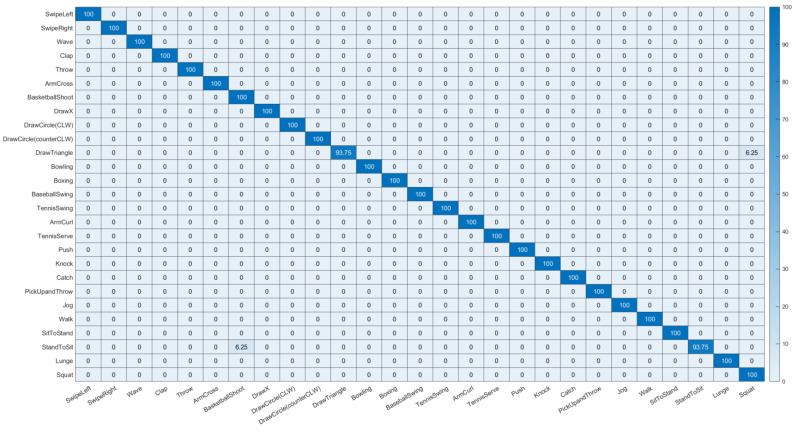
Confusion chart for UTD-MHAD dataset using joints + velocity + CCA.

We compared the proposed method with state-of-the-art HAR methods using the UTD-MHAD dataset, as shown in Table 3. The proposed method showed better results than the prior studies. We also reported the confusion chart for the best experimental setting to show the individual recognition results for individual classes. Figure 9 depicts the confusion chart for the joints + velocity + CCA setting with the UTD-MHAD dataset. The proposed method achieved minimum detection accuracies of approximately 93.75% for actions *DrawTriangle* and *StandToSit*, whereby 6.25% of cases were detected as *Squat* and *BasketballShoot*, respectively. For other action classes, the proposed method could correctly detect all test samples.

### 5.5. Performance Evaluations and Comparisons on the MSR-Action3D Dataset

To show the effectiveness and robustness of the proposed method, we determined the recognition precision, recall, accuracy, and F1-score on another human action dataset called MSR-Action3D using the same settings as described in Section 5.4. Table 4 lists the recognition precision, recall, accuracy, and F1-score on the MSR-Action3D dataset. The proposed method achieved approximately 94.18%, 89.09%, 94.91%, and 95.64% recognition accuracies for original joints, velocity, joints + velocity, and joints + velocity + CCA, respectively. The performance improvements with the aggregation of joints, velocity, and CCA were approximately 1.46%, 6.55%, and 0.73% with respect to joints, velocity, and joints + velocity, respectively.

**Figure 10:**
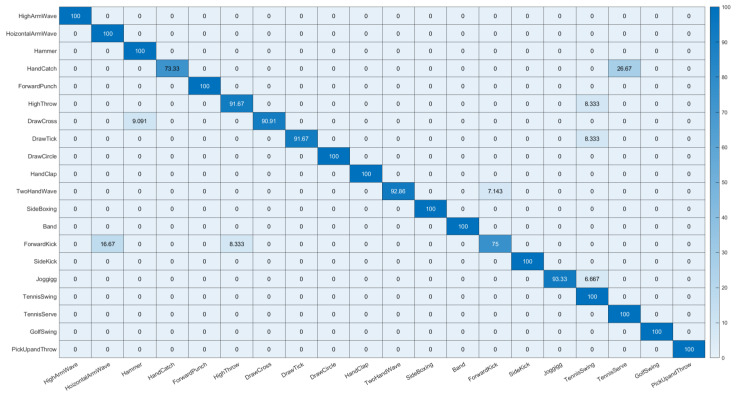
Confusion chart for MSR-Action3D dataset using joints + velocity + CCA.

We compared the proposed method with prior studies which used the MSR-Action3D dataset as listed in Table 5. Again, the proposed method worked much better for HAR using the MSR-Action3D dataset than the state-of-the-art methods. We further provide a confusion chart to show the detection performance of the proposed method on each action class, as shown in Figure 10. We used the joints + velocity + CCA setting to compute the confusion chart. The proposed best setting in the MSR-Action3D dataset obtained a minimum accuracy of approximately 73.33% and 75.00% for *HandCatch* and *ForwardKick* actions. For action *HandCatch,* 26.67% of test samples were incorrectly detected as the *TennisServe* action class. For action *ForwardKick,* 16.67% and 8.33% of test samples were incorrectly detected as the *HorizontalArmWave* and *HighThrow* action classes. For other action classes, the recognition accuracies were above 90% for the proposed method with the best setting.

### 5.6. Effects of Criss-Cross Attention on Recognitoion Performance

We carefully chose the parameter set to train and test the proposed method. We integrated spatial and temporal attention and set the value of k as 20 to evaluate the performance in Section 5.3 and Section 5.4. We investigated the recognition results for spatial and temporal attention separately to show the effects of combined spatial and temporal attention, as shown in Table 6. The combination of spatial and temporal attention ensured better results of approximately 99.53% and 95.64% for the UTD-MHAD and MSR-Action3D datasets than separately trained models.

We also examined the trend of recognition accuracies for different k-values on the UTD-MHAD dataset to choose the best one. The proposed method achieved accuracies of 97.67%, 98.37%, 99.53%, 98.60%, and 97.45% for k-values of 10, 15, 20, 25, and 30, respectively. The proposed deep learning model ensured better results for a k-value of 20. Consequently, we chose the value of k as 20 for the overall evaluation of the proposed method.

### 5.7. Network Architecture and Complexity Analysis

For better understanding and explanation, we report the architectural overview of the proposed deep learning model for the combined model with joints, velocity, and CCA. Table 7 lists the names of the modules, components in each module, input/output, and parameters. We present 2D normalization as Norm2D, 2D convolution as Conv2D, k-nearest neighbor as k-NN, and 2D adaptive max pooling as adaptMaxPool2D. The total number of parameters of the proposed model was approximately 1,300,103. We computed the average execution time for the test samples for the best setting, and it required 0.011 s to recognize an action. The proposed model required 831,190,224 floating-point operations per second to accomplish the recognition.

## 6. Conclusions

Human action recognition has been studied widely for its multitude of applications in the fields of computer vision and pattern recognition. Currently, it has become very important to introduce an effective and robust human action recognition system owing to the pandemic, which has led to the avoidance of intimate contact. In this article, we addressed a new method for human action recognition by designing a novel deep learning model with criss-cross attention and edge convolution layers, incorporating the local and global relationships among the joints and bones to capture spatial and temporal information. The attention technique was applied in the spatial and temporal directions to focus more on the intra- and inter-frame changes. The proposed model is updated dynamically after each layer by recomputing the graph which changes the k-nearest neighbor of a joint from layer to layer. This model also learns the way of constructing k-nearest neighbor graphs at each layer rather than operating in a fixed graph from the beginning. We evaluated the proposed model on two different datasets with different parameter settings to show its effectiveness and efficiency. The dynamic edge convolutional neural network showed better results than state-of-the art methods. This study focused only on single-person action recognition, in which there were no occlusions. In the future, we will evaluate action datasets with multiple persons interacting interpersonally or with a machine. We will also consider point-cloud human action datasets for action recognition to provide easy, smart, and effective interaction in the metaverse world.

## Figures and Tables

**Table 1:** State-of-the-art study descriptions.

Methods	Year	Features/Classifiers	Dataset
Tradtional machine learning-based	2017	MIMTL [22]	MSR-Action3D
	2013	Cov3DJ, SVM [23]	MSR-Action3D
	2012	HOJ3D, LDA, HMM [24]	MSR-Action3D
	2012	EigenJoints, NBNN [25]	MSR-Action3D
	2014	Lie group, SVM [26]	MSR-Action3D
	2019	Bag of poses, K-means, SVM, ELM [27]	MSR-Action3D, UTD-MHAD
	2013	LDS, Discriminative metric learning, MKL [28]	MSR-Action3D
Deep Learning-based	2016	SOS, ConvNets [29]	UTD-MHAD
	2018	JTM, ConvNets [30]	UTD-MHAD
	2020	TPSMMs, ConvNets [31]	UTD-MHAD
	2021	STIF, MobileNetV2, DenseNet121, ResNet18 [32]	UTD-MHAD
	2021	Joint location, Edge motion, SEMN [33]	UTD-MHAD
	2019	GCN, LSTM [34]	MSR-Action3D, UTD-MHAD
	2021	Attention, ST-GCN [35]	UTD-MHAD
	2021	AMV-GCN [36]	UTD-MHAD
	2022	ST-GCN and ResNeXt [37]	UTD-MHAD
	2022	Key nodes selection, GCN [38]	MSR-Action3D, UTD-MHAD
	2022	HP-DMI, ST-GCN, STJD, SVM [39]	MSR-Action3D, UTD-MHAD

**Table 2:** Performance evaluation of the proposed HAR method on the UTD-MHAD dataset.

Method	Precion	Recall	Accuracy	F1-Score
Joints (Base)	98.92%	98.84%	98.84%	98.83%
Velocity	97.02%	96.53%	96.51%	96.52%
Joints + Velocity	99.13%	99.07%	99.07%	99.07%
**Joints + Velocity + CCA**	**99.56%**	**99.54%**	**99.53%**	**99.54%**

**Table 3:** Performance comparisons of HAR methods on the UTD-MHAD dataset.

Method	Precision	Recall	Accuracy	F1-Score
Agahian et al. [27], 2019	95.75%	95.37%	95.30%	95.33%
Hou et al. [29], 2016	89.25%	87.04%	86.97%	86.94%
Wang et al. [30], 2018	-	-	87.45%	-
Chen et al. [31], 2020	*-*	*-*	88.10%	*-*
Tasnim et al. [32], 2021	*-*	*-*	95.29%	*-*
Wang et al. [33], 2021	*-*	*-*	95.59%	*-*
Zhao et al. [34], 2019	*-*	*-*	92.10%	*-*
Ahmad et al. [35], 2021	*-*	*-*	99.50%	*-*
Liu et al. [36], 2021	*-*	*-*	95.11%	*-*
Liu et al. [37], 2022	*-*	*-*	80.23%	*-*
Zhang et al. [38], 2022	94.69%	94.17%	94.19%	94.00%
Cha et al [39], 2022	-	-	96.30%	-
Wu et al. [41], 2022	95.41%	94.89%	94.85%	94.86%
**Proposed (Joints + Velocity + CCA)**	**99.56%**	**99.54%**	**99.53%**	**99.54%**

**Table 4:** Performance evaluation of the proposed HAR method on the MSR-Action3D dataset.

Method	Precision	Recall	Accuracy	F1-Score
Joints (Base)	95.20%	93.57%	94.18%	93.66%
Velocity	89.79%	87.98%	89.09%	88.41%
Joints + Velocity	95.33%	94.60%	94.91%	94.52%
**Joints + Velocity + CCA**	**96.12%**	**95.44%**	**95.64%**	**95.47%**

**Table 5:** Performance comparisons of HAR methods on the MSR-Action3D dataset.

Method	Precision	Recall	Accuracy	F1-Score
Yang et al. [22], 2017	-	-	93.63%	-
Hussein et al. [23], 2013	-	-	90.53%	-
Xia et al. [24], 2012	-	-	78.97%	-
Yang et al. [25], 2012	-	-	83.30%	-
Vemulapalli et al. [26], 2014	-	-	92.46%	-
Agahian et al. [27], 2019	92.65%	91.76%	91.90%	91.61%
Zhao et al. [34], 2019	-	-	94.50%	-
Zhang et al. [38], 2022	95.79%	95.73%	94.81%	95.53%
Wu et al. [41], 2022	95.41%	95.30%	95.18%	95.24%
**Proposed (Joints + Velocity + CCA)**	**96.12%**	**95.44%**	**95.64%**	**95.47%**

**Table 6:** Performance of different attention mechanisms of the proposed method on UTD-MHAD and MSR-Action3D dataset.

Method	UTD-MHAD	MSR-Action3D
Joints + Velocity + CCA (Spatial)	99.07%	95.27%
Joints + Velocity + CCA (Temporal)	99.30%	94.91%
**Joints + Velocity + CCA (Spatial + Temporal)**	**99.53%**	**95.64%**

**Table 7:** Details of each module in proposed model for HAR.

Module	Component	[Input]→[Output]	Parameter
Attention Module (Joints)	Norm2D, Conv2D, ReLU	[20 × 20 × 3]→[20 × 20 × 64]	262
	Conv2D, ReLU	[20 × 20 × 64]→[20 × 20 × 64]	4160
	CCA-Spatial (Conv2D, Conv2D, Conv2D, Softmax)	[20 × 20 × 64]→[20 × 20 × 64]	504
	CCA-Temporal (Conv2D, Conv2D, Conv2D, Softmax)	[20 × 20 × 64]→[20 × 20 × 64]	504
Attention Module (Velocity)	Norm2D, Conv2D, ReLU	[20 × 20 × 3]→[20 × 20 × 64]	262
	Conv2D, ReLU	[20 × 20 × 64]→[20 × 20 × 64]	4160
	CCA-Spatial (Conv2D, Conv2D, Conv2D, Softmax)	[20 × 20 × 64]→[20 × 20 × 64]	504
	CCA-Temporal (Conv2D, Conv2D, Conv2D, Softmax)	[20 × 20 × 64]→[20 × 20 × 64]	504
EdgeConv Module	k-NN Graph, Conv2D, Norm2D, LeakyReLU, Max	[20 × 20 × 64]→[20 × 20 × 64]	8320
	k-NN Graph, Conv2D, Norm2D, LeakyReLU, Max	[20 × 20 × 64]→[20 × 20 × 64]	8320
	k-NN Graph, Conv2D, Norm2D, LeakyReLU, Max	[20 × 20 × 64]→[20 × 20 × 128]	16,640
	k-NN Graph, Conv2D, Norm2D, LeakyReLU, Max	[20 × 20 × 128]→[20 × 20 × 256]	66,048
Classification Module	AdaptMaxPool2D, Conv2D, Norm2D, ReLU	[20 × 20 × 512]→[1 × 20 × 512]	787,968
	Conv2D, Norm2D, ReLU	[1 × 20 × 512]→[1 × 20 × 512]	263,680
	AdaptMaxPool2D, Linear, ReLU, Dropout	[1 × 20 × 512]→[1 × 256]	131,328
	Linear, Softmax	[1 × 256]→[1 × 27]	6939
**Total**			**1,300,103**

## Data Availability

Not applicable.
